# Comparison of intraoral biofilm reduction on silver-coated and silver ion-implanted stainless steel bracket material: Biofilm reduction on silver ion-implanted bracket material

**DOI:** 10.1007/s00056-018-00165-3

**Published:** 2018-12-10

**Authors:** Viktoria Meyer-Kobbe, Katharina Doll, Meike Stiesch, Rainer Schwestka-Polly, Anton Demling

**Affiliations:** 1 Department of Orthodontics, Hannover Medical School, Carl-Neuberg-Str. 1, 30625 Hannover, Germany; 2 Department of Prosthetic Dentistry and Biomedical Materials Science, Hannover Medical School, Carl-Neuberg-Str. 1, 30625 Hannover, Germany

**Keywords:** Plasma immersion ion implantation and deposition, Silver ions, Biofilms, In situ study, Confocal laser scanning microscopy, Orthodontic bracket material, Plasma immersion ion implantation and deposition, Silberionen, Biofilme, In-situ-Studie, Konfokale Laser-Scanning-Mikroskopie, Kieferorthopädisches Bracketmaterial

## Abstract

**Purpose:**

The objective of this in situ study was to quantify the intraoral biofilm reduction on bracket material as a result of different surface modifications using silver ions. In addition to galvanic silver coating and physical vapor deposition (PVD), the plasma immersion ion implantation and deposition (PIIID) procedure was investigated for the first time within an orthodontic application.

**Materials and methods:**

An occlusal splint equipped with differently silver-modified test specimens based on stainless steel bracket material was prepared for a total of 12 periodontally healthy patients and was worn in the mouth for 48 h. The initially formed biofilm was fluorescently stained and a quantitative comparative analysis of biofilm volume, biofilm surface coverage and live/dead distribution of bacteria was performed by confocal laser scanning microscopy (CLSM).

**Results:**

Compared to untreated stainless steel bracket material, the antibacterial effect of the PIIID silver-modified surface was just as significant with regard to reducing the biofilm volume and the surface coverage as the galvanically applied silver layer and the PVD silver coating. Regarding the live/dead distribution, however, the PIIID modification was the only surface that showed a significant increase in the proportion of dead cells compared to untreated bracket material and the galvanic coating.

**Conclusions:**

Orthodontic stainless steel with a silver-modified surface by PIIID procedure showed an effective reduction in the intraoral biofilm formation compared to untreated bracket material, in a similar manner to PVD and galvanic silver coatings applied to the surface. Additionally, the PIIID silver-modified surface has an increased bactericidal effect.

## Zusammenfassung

**Zielsetzung:**

Das Ziel der vorliegenden In-situ-Studie war die Quantifizierung der intraoralen Biofilmreduktion auf Bracketmaterial nach unterschiedlichen Oberflächenmodifikationen mittels Silberionen. Zusätzlich zur galvanisch aufgebrachten Silberbeschichtung und der Beschichtung mittels PVD („physical vapor deposition“) wurde das Verfahren der PIIID („plasma immersion ion implantation and deposition“) erstmals in der kieferorthopädischen Anwendung untersucht.

**Material und Methodik:**

Eine Okklusionsschiene, bestückt mit unterschiedlich silbermodifizierten Probekörpern aus Edelstahl-Bracketmaterial, wurde für insgesamt 12 parodontal gesunde Probanden hergestellt und für 48 h im Mund getragen. Der initial gebildete Biofilm wurde fluoreszenzgefärbt und eine quantitative, vergleichende Analyse des Biofilmvolumens, der bakteriellen Flächenbelegung und der Lebend/Tot-Verteilung der Bakterien wurde mittels konfokaler Laser-Scanning-Mikroskopie (CLSM) durchgeführt.

**Ergebnisse:**

Im Vergleich zu unbehandeltem Edelstahl-Bracketmaterial war die PIIID-silbermodifizierte Oberfläche hinsichtlich der Reduktion des Biofilmvolumens und der bakteriellen Flächenbelegung ebenso signifikant antibakteriell wirksam wie die galvanisch aufgebrachte Silberbeschichtung und die PVD-Silberbeschichtung. In Bezug auf die Lebend/Tot-Verteilung wies die PIIID-Modifikation jedoch als einzige Oberfläche eine signifikante Zunahme des Tot-Bakterienanteils im Vergleich zu unbehandeltem Bracketmaterial und der galvanischen Silberbeschichtung auf.

**Schlussfolgerungen:**

Kieferorthopädisch verwendeter Edelstahl, dessen Oberfläche mittels des PIIID-Verfahrens silbermodifiziert wurde, zeigt im Vergleich zu unbehandeltem Bracketmaterial eine wirksame Reduktion der intraoralen Biofilmbildung, ebenso wie die oberflächlich aufgetragene PVD- und galvanische Silberbeschichtung. Darüber hinaus hat die PIIID-silbermodifizierte Oberfläche einen verstärkten bakteriziden Effekt.

## Introduction

The formation of biofilm in the oral cavity is one of the major problems in dentistry [13, 27, 58]. Microorganisms trigger caries or periodontopathies, which represent the main conditions that require therapy in the field of dentistry [16, 37, 38]. In addition, it has been shown that periodontal disease is closely associated with systemic diseases, such as arteriosclerotic changes in the blood-conducting vessels [23, 41, 42, 54, 57, 65].

Daily thorough cleaning of the teeth (multiple times per day) can counteract the formation of biofilm but does not eliminate it completely [33]. Immediately after cleaning the teeth, a 0.1–1 µm-thick pellicle is formed from adsorbed proteins on the enamel [34]. Within a few hours, this pellicle enables the adherence of early bacterial colonizers (e. g. *Streptococcus salivarius, Streptococcus oralis*) [34]. The plaque continues to grow by accumulation of late colonizers (e. g. *Aggregatibacter actinomycetemcomitans, Treponema denticola*), thereby forming a three-dimensional structure. The components of a mature biofilm are 60–70 vol.% bacteria, embedded in a matrix of extracellular polysaccharides [34]. If a plaque has reached this stage, it can no longer be eliminated by self-cleaning effects in the oral cavity. The bacteria produce organic acids from supplied carbohydrates. A bacterial shift toward an acidic anaerobic environment occurs and the organic acids diffuse into the enamel, thereby releasing calcium and phosphate ions from the crystal lattice. These decalcifications of enamel are subsequently visible as “white spot lesions”. In the event of a prolonged acidic environment and progressive demineralization processes, the initial enamel caries lead to dentin caries [12, 34, 36].

Orthodontic appliances, which are used to treat up to 58% of children and adolescents in Germany [12], represent a particular problem regarding teeth cleaning. Due to their geometry, fixed braces in particular have various recesses that are difficult to clean. This impacts oral hygiene and reduces the self-cleaning effect of the teeth [14]. It can subsequently result in increased plaque accumulation, which can, in turn, cause gingivitis and increased probing depths [18, 20]. As mentioned above, mature plaque has a high acidogenic potential and increases the risk of enamel demineralization and the formation of caries in the area around the bracket [5, 60]. As part of an increasing awareness of health-related issues and for reasons of clinical necessity, preventive treatment concepts are gaining increasing significance [25].

Despite numerous and extensive prevention concepts (e. g. bracket ligating materials containing fluoride [47], enamel sealing in the immediate proximity of the bracket [4, 24, 32], polytetrafluorethylene-coated bracket surfaces [19, 29]), increased plaque accumulation and the subsequent appearance of “white spot lesions” can still be clinically proven [56, 64]. For this reason, additional approaches are required, which would ideally prevent the formation of the initial intraoral biofilm on brackets from the outset. This could be achieved by antibacterial properties of the orthodontic appliances themselves—an approach that is already being pursued in the field of dental implant research [17, 22]. For this purpose, silver is a material that is frequently examined for bracket appliances and implants in the field of dentistry. The precious metal exhibits an antibacterial effect through inhibition of enzymes that are involved in the respiratory chain, thereby disrupting the bacterial metabolism [53]. There are also studies that show that silver particles prevent the replication capability of the deoxyribonucleic acid (DNA) of microorganisms [26]. In vitro investigations of a physical vapor deposition (PVD) coated silver-platinum layer in combination with a subsequent heat treatment, a reduced biofilm adherence of *Streptococcus mutans* and *Aggregatibacter actinomycetemcomitans* with simultaneous good biocompatibility was observed [55]. Similar effects were also demonstrated for an alloy of silver and gold nanoparticles, a silver titanium dioxide coating and a silver layer enclosed by plasmapolymers [6, 7, 31, 67]. In addition, an animal study showed that silver-coated surfaces of orthodontic appliances have an antibacterial effect on *Streptococcus mutans *without additional oral hygiene and patient compliance, thereby reducing the risk of caries [48]. However, there is a decrease in the silver particles released as the orthodontic treatment duration with fixed appliances increases [48]. In addition, the low abrasion resistance of coatings applied on top of the surface poses a problem. The surface layer can be quickly worn down under the prevailing oral conditions [29]. The antibacterial effect of a coating only applied on top of the surface is therefore non-permanent as it loses its effect due to either partial delamination or surface abrasions.

A possibility for increasing the abrasion resistance of silver-modified surfaces is to use the plasma immersion ion implantation and deposition (PIIID) procedure. This technique is a vacuum process during which a metallic sacrificial cathode (here the silver target) is heated until it reaches red heat. By applying negative pulsed high-voltage potentials, silver ions are released from the cathode and accelerated in the direction towards the material (here the bracket material) submerged in a plasma. The high energy metal ions are implanted in the surface of the material [1, 45, 49]. According to manufacturer’s data, an additional silver top layer is built up on the surface. Through a heat treatment following the implantation process, diffusion of the silver ions into deeper lattice structures of the bracket material are achieved and the penetration depth is increased [49]. In theory, the PIIID procedure promises improved abrasion stability of the implanted silver ions compared to silver layers only applied on top of the surface. As the antibacterial effect of silver-implanted bracket material has not yet been described in the field of orthodontic appliances, the objective of this clinical study is the initial investigation of plaque accumulation on PIIID-modified surface. After being worn in the mouth for 48 h, the plaque accumulation was examined for untreated bracket material as well as PVD and galvanically silver-coated bracket material and compared with the antimicrobial effect of PIIID silver-implanted surface. It was analyzed if the null hypothesis, stating no statistical significant differences (*p* ≤ 0.05) between the four various groups, could be rejected.

## Materials and methods

### Subject selection

There is a positive ethics vote in place (ethics vote no. 4347 “Biofilm formation on fixed orthodontic appliances and microimplants”) from the Hannover Medical School for the conducted study. Twelve periodontally healthy subjects (6 women and 6 men) were enrolled in the study. This was ensured by an initial periodontal screening process. During the screening process, the modified approximal plaque index (API) according to Lange et al., the modified sulcus bleeding index (SBI) according to Lange and the probing depths (PD) were measured [34]. The probing depth measurements were performed in a randomized manner (block randomization with a block size of 12) in the first and third or second and fourth quadrants, on the middle front teeth, the first premolars and the first molars in each case. The probing depths should not be greater than 4 mm and the plaque index not higher than 25%. Pregnancy, general illness, smoking, a removable tooth replacement and antibiotic therapy that was started less than 6 weeks before the start of the study constituted additional exclusion criteria. The patients were informed about the study in advance by an information sheet and an explanatory discussion. The patients’ consent was obtained in writing in the form of a consent form. The data analyses were anonymized.

### Occlusal splint design

**Fig. 1:**
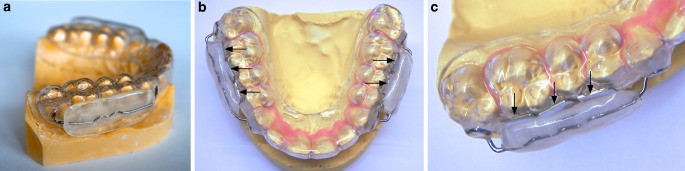
**a** Occlusal splint with vestibular plastic shield-like construction and **b**, **c** fixed test specimens in premolar and molar region (*arrows*)

Dental casts of the upper jaw were made for the 12 subjects (Alginoplast®, Kulzer GmbH, Hanau, Germany). On the resulting plaster model, an occlusal splint made of plastic was manufactured using the thermoplastic deep drawing procedure (Erkodur, Erkodent® Erich Kopp GmbH, Pfalzgrafenweiler, Germany). A barrier was then fitted in the posterior region to hold the cheek and tongue. This barrier resembled a shield, such as those used for the function regulator of Fränkel [39]. Using sprinkle and spray technology, clear plastic (Orthocryl®, Dentaurum GmbH & Co. KG, Ispringen, Germany) was applied to a wire retainer (Fino, DT & Shop GmbH, Bad Bocklet, Germany), which was connected to the occlusal splint, and polymerized. This resulted in a gap of a few millimeters between the deep-drawn occlusal splint and the shield-like construction (Fig. 1a).

### Modification and characterization of test specimens

Test specimens made from commonly used bracket material with dimensions of approx. 6.5 × 7 × 2 mm (Forestadent Bernhard Förster GmbH, Pforzheim, Germany) were used for the study. The composition of the bracket material was initially analyzed using energy-dispersive X‑ray (EDX) analysis (scanning electron microscope, Tescan Vega 3 with EDX, Brno, Czech Republic). The test specimens were then fixed to a carrier plate by two laser weld points, grounded using a grinding machine (belt grinder, Timesavers International B.V., Goes, The Netherlands) and reduced to the required thickness of 0.5 mm. The samples were polished to a high finish using universal polishing paste (Universal Polishing Paste, Ivoclar Vivadent AG, Schaan, Liechtenstein). The PIIID procedure, PVD coating (both MAT Dresden, Dresden, Germany) and galvanic coating (Herbst Galvano GmbH, Schnaittach, Germany) were used for surface modification of the test specimens with silver ions. According to the manufacturers, a silver target of 99.9% silver (Ag) was used in each case. The resulting roughness of the silver-modified and unmodified test specimens was measured using confocal laser-scanning microscopy (CLSM, Keyence VK-X100 series, Keyence Deutschland GmbH, Neu-Isenburg, Germany). The various surface modifications were illustrated by pictures of cross-sections at a magnification of 1000 × (Axio Scope.A1 metallography microscope, Carl Zeiss MicroImaging GmbH, Göttingen, Germany). For the PIIID an EDX analysis was carried out on the additionally deposited silver surface top layer. In addition, as a theoretical proof, the penetration depth of the silver ions into the bracket material was calculated using a simulation of the transport of ions in matter (TRIM; http://www.srim.org/).

### Insertion of the prepared splints for 48 h

Per splint, an untreated control as well as the different silver-modified test specimens were placed in the first and second quadrants in the premolar and molar region in each case (Fig. 1b). The samples that had been silver-modified were divided into two groups. In group A, a control and PVD were combined, while in group B, a control, PIIID and the galvanic coating were grouped together. Within both groups, the position of the platelet was rotated on the splint. In addition, the quadrant selection was randomized in order to prevent any falsification of the data as a result of potential localizing influences in the oral cavity. Using a flowable composite (Tetric EvoFlow, Ivoclar Vivadent AG, Schaan, Liechtenstein) as well as a temporary cement (TempBond™, Kerr GmbH, Biberach, Germany), the test specimens were placed on the splints and degreased with alcohol. The adhesive joints on the splints were sandblasted in advance using aluminum oxide (28–70 µm) (Basic quattro IS, Renfert GmbH, Hilzingen, Germany) in order to improve the adherence of the composite to the platelet. The splints with the samples were inserted on the test subjects for 48 h. Oral hygiene was suspended for the duration of the examination period. In addition, the patients were not allowed to consume alcohol. After wearing the occlusal splint for 48 h, the samples were removed from the carrier splints while maintaining the biofilm that had formed.

### Microscopic analysis of biofilm

Using the LIVE/DEAD® *Bac*Light^TM^ Bacterial Viability Kit (Thermo Fisher Scientific, Braunschweig, Germany), the plaque deposits were fluorescently stained according to the manufacturer’s protocol and fixed with 2.5% glutaraldehyde (Carl Roth GmbH + Co. KG, Karlsruhe, Germany). A phosphate-buffered saline solution (Biochrom GmbH, Berlin, Germany) was used to store the stained samples at 4 °C. This enabled the initial intraoral biofilm formation to be assessed under close-to in vivo conditions. Three-dimensional images of the biofilm were taken by CLSM (SP2, Leica Microsystems GmbH, Wetzlar, Germany). For each test specimen, five defined positions were microscopically examined at a magnification of 10 × and 63 ×. The biofilm volume per test specimen and the live/dead distribution were quantified using the Imaris software package (Imaris x64 6.2.1, Bitplane AG, Zurich, Switzerland). In addition, a representative three-dimensional reconstruction of the biofilm was carried out. The biofilm surface coverage per test specimen was determined using the Leica LAS AF Lite and the ImageJ software (Leica LAS AF Lite, Leica Microsystems GmbH, Wetzlar, Germany; ImageJ, Wayne Rasband, National Institutes of Health, Bethesda, MD, USA, http://imagej.nih.gov/ij/).

### Statistical analysis

The statistical analysis was performed using the GraphPad Prism software (GraphPad Software, Inc., La Jolla, CA, USA). Data were tested for Gaussian distribution using the D’Agostino-Pearson Omnibus normality test. The unmodified controls inserted in different quadrants were compared using the paired t‑test. Comparison of unmodified controls with the silver-modified samples was done using the Friedman test with Dunett’s correction for multiple comparisons. All statistical analyses were carried out in a two-sided manner at a significance level of *p* ≤ 0.05.

## Results

### Test subjects

From a total of 12 test subjects, 11 test subjects aged between 20 and 34 years old (24.8 ± 3.8 years), weighing between 57 and 98 kg (71.3 ± 14.3 kg) and with a height of between 165 and 200 cm (176.4 ± 10.1 cm) could be included in this study in compliance with the trial protocol. The periodontal screening performed at the start revealed an API of 14.7 ± 7.8%, a SBI of 10.7 ± 7.7% and a PD of 1.6 ± 0.2 mm.

### Characterization of the silver-modified test specimens

**Fig. 2:**
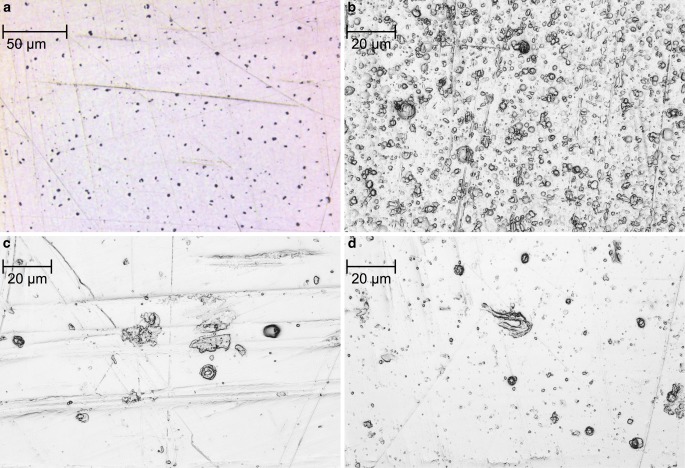
Surface roughness imaged by confocal laser scanning microscopy (CLSM) on **a** unmodified bracket material, **b** galvanic coating, **c** physical vapor deposition (PVD) coating and **d** surface modified by plasma immersion ion implantation and deposition (PIIID) procedure

**Fig. 3:**
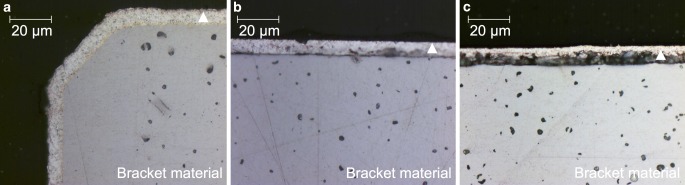
Cross sections of the silver surface layer on the bracket material , marked with *triangles*: **a** galvanic coating adhered directly to bracket material; **b** physical vapor deposition (PVD) coating and **c** surface modified by plasma immersion ion implantation and deposition (PIIID) procedure with a porous interlayer between silver coating and bracket material

**Fig. 4:**
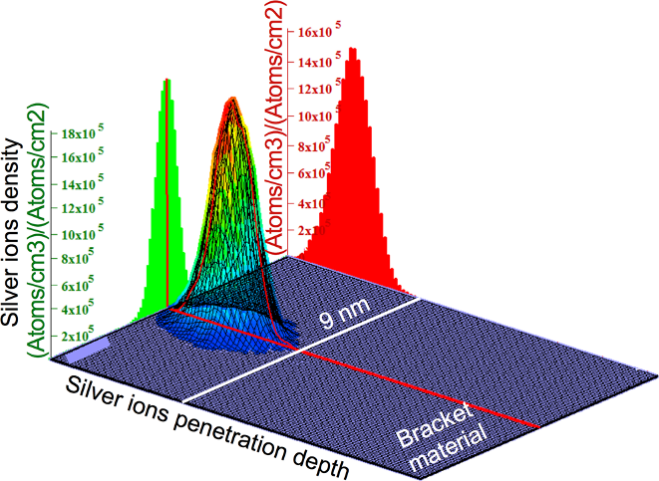
For plasma immersion ion implantation and deposition (PIIID), the transport of ions in matter (TRIM) simulation illustrates the silver ions density in dependence on the calculated penetration depth of silver ions into the bracket material

Initially, an EDX analysis was performed on the unmodified bracket material, which was used as control group for all additional tests. According to this analysis, the material consisted of 67.91 wt % iron (Fe), 16.64 wt % chromium (Cr), 8.56 wt % manganese (Mn), 4.05 wt % molybdenum (Mo), 2.42 wt % carbon (C) and 0.42 wt % silicon (Si). This corresponds to austenitic, nickel-free stainless steel with the material number 1.4456 [66]. After the surface modifications of the bracket material with silver, the surface roughness was determined in order to ensure consistent initial conditions. The average surface roughness (Ra) for the untreated bracket material was Ra = 0.04 µm, for the galvanic coating Ra = 0.12 µm, for the PVD coating Ra = 0.08 µm and for the PIIID procedure Ra = 0.06 µm (Fig. 2). Therefore, all surfaces had a roughness below the threshold value Ra = 0.2 µm indicated in the literature [8, 52, 62]. The galvanic coating adhered directly to the bracket material. According to data from the manufacturer, the thickness of the galvanically applied silver layer was 5 µm (Fig. 3a). For PVD, the cross-section revealed a porous interlayer between the silver coating and the bracket material. According to producer’s information, the PVD coating should have a total thickness of 1 µm, but the picture of the cross-section showed a thicker layer (Fig. 3b). For PIIID, the cross-section revealed also an even more pronounced porous interlayer between the silver top layer and the bracket material. Corresponding to manufacturer’s data, the supplementary deposited silver top layer was 1 µm (Fig. 3c). The EDX analysis of the additionally applied silver top layer of the PIIID revealed an elemental composition of 99.2 wt % Ag, 0.7 wt % Fe and 0.20 wt % Cr. According to manufacturer’s data, the penetration depth of the implanted silver ions into the bracket material was approx. >1 µm. However, according to the TRIM simulation performed for PIIID, it must be assumed that the calculated penetration depth of the silver ions was only up to 9 nm (Fig. 4).

### No differences between control groups of the first and second quadrant

**Fig. 5:**
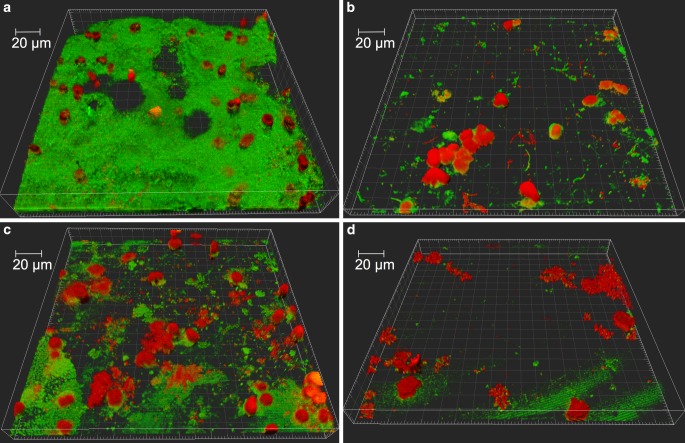
Representative three-dimensional (3D) reconstructions of confocal laser scanning microscopy (CLSM) gained raw data at a magnification of 63 × depicted the accumulating biofilm on **a** unmodified bracket material, **b** galvanic coating, **c** physical vapor deposition (PVD) coating and **d** surface modified by plasma immersion ion implantation and deposition (PIIID) procedure

**Fig. 6:**
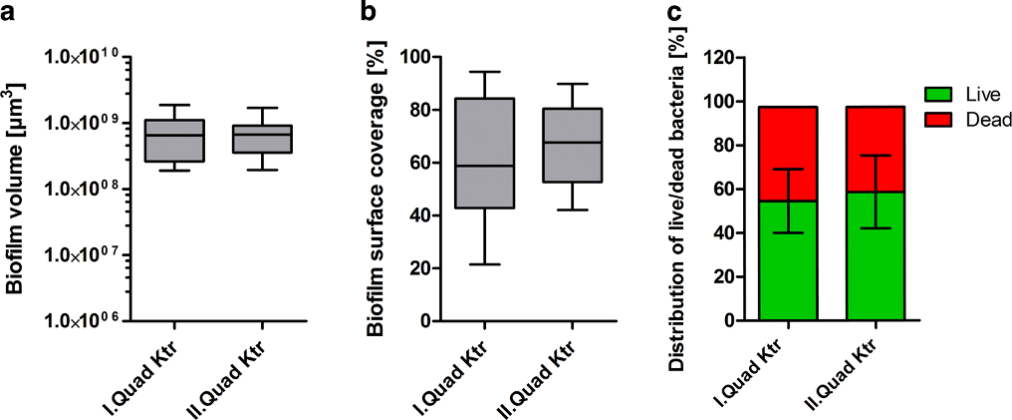
Box-plot diagram of average **a** biofilm volume, **b** biofilm surface coverage and **c** distribution of live/dead bacteria on control group of the first and second quadrants

After 48 h of wearing the occlusal splint fitted with the test specimens, the attached biofilm was fluorescently stained and assessed by CLSM. Representative three-dimensional (3D) reconstructions of the accumulated plaque are shown in Fig. 5 and reveal that the biofilms consist of bacteria and a few human cells. Initially, the biofilm formation on the control samples in the first and second quadrants were compared with one another. For the biofilm volume, the surface coverage and the live/dead distribution, there were no statistical significant differences between the two quadrants. The average biofilm volume in the first quadrant was 7.46 × 10^8^ ± 5.31 × 10^8^ µm^3^ and in the second quadrant 7.01 × 10^8^ ± 4.17 × 10^8^ µm^3^ (Fig. 6a). The average percentage surface coverage for the controls in the first quadrant was 61.35 ± 23.87% and in the second quadrant 67.45 ± 15.91% (Fig. 6b). The average live/dead proportion in the first quadrant was 55/45% and was therefore similar to the second quadrant with 59/41% (Fig. 6c). For the subsequent evaluation of the silver-modified test specimens, the two individual control groups were therefore consolidated into one joint comparison group.

### Reduced biofilm formation on silver-modified test specimens

**Fig. 7:**
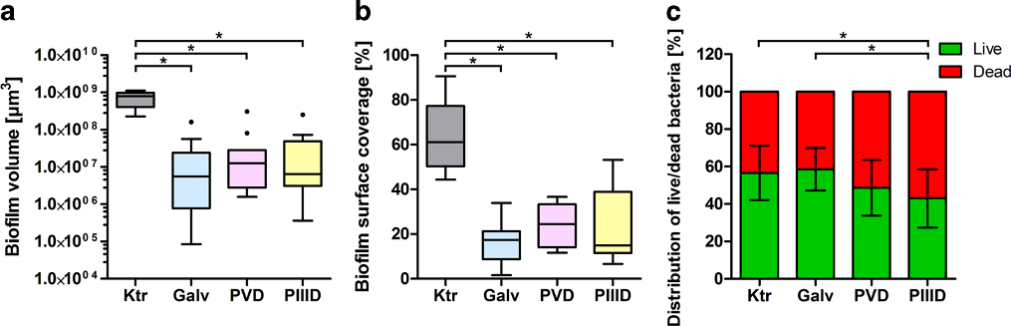
Box-plot diagram of average **a** biofilm volume, **b** biofilm surface coverage and **c** distribution of live/dead bacteria on control group and the various surface modifications. Statistical significant differences with a significance level of *p* ≤ 0.05 were marked with an *asterisk*. *Ktr* control, *Galv* galvanic, *PVD* physical vapor deposition, *PIIID* plasma immersion ion implantation and deposition

The null hypothesis, stating no statistical significant differences with a significance level of *p* ≤ 0.05 between the four various groups, could be rejected in this study. The comparison of the biofilm formation on the different test specimens after being worn for 48 h revealed a significant reduction in plaque on the silver-modified surfaces. This was quantified with the parameters of biofilm volume, biofilm surface coverage and live/dead distribution. The biofilm volume per test specimen for the control was 7.24 × 10^8^ ± 3.11 × 10^8^ µm^3^. For the galvanically applied silver coating, the biofilm volume decreased to 2.62 × 10^7^ ± 4.81 × 10^7^ µm^3^, for the PVD coating to 4.44 × 10^7^ ± 9.06 × 10^7^ µm^3^ and for the PIIID procedure to 3.82 × 10^7^ ± 7.53 × 10^7^ µm^3^. The reduction of the biofilm volume compared to the control was statistically significant for all surface modifications. No statistical differences between the individual procedures were observed (Fig. 7a). The percentage surface coverage per test specimen was 64.40 ± 15.73% for the unmodified control and decreased to 16.97 ± 9.33% for the galvanic silver surface, to 23.81 ± 9.76% for the PVD coating and to 23.63 ± 15.52% for the PIIID-modified surface. As with the comparison of the biofilm volume, statistical significant differences were detected between the control and the surface layer modifications, but not between the individual modifications (Fig. 7b). The quantification of the biofilms live/dead distribution was 57/43% for the unmodified control, 59/41% for the galvanic silver surface, 49/51% for the PVD coating and 43/57% for the PIIID-modified surface. For the PIIID-modified surfaces, a statistical significant increase in dead bacteria was detected compared to the control, but also to the galvanically coated test specimens. Between all other groups, no statistical significant differences could be detected (Fig. 7c).

## Discussion

The objective of this study was to determine whether implanting silver ions into the surface of stainless steel bracket material using the PIIID procedure has an antibacterial effect on accumulating oral plaque and how comparable this effect is to untreated bracket material, a PVD and galvanic silver coating. In the field of orthodontics, this is the first study to use the PIIID procedure—which is already used in other branches of the medical engineering industry [50].

In order to test the PIIID procedure under normal, periodontally healthy oral flora conditions, the test subjects underwent a periodontal screening examination in advance, which revealed no unusual values regarding API, SBI and PD. In addition, the test subjects did not present any other risk factors that could adjust the composition of the microbial flora, such as pregnancy or smoking [9, 15, 30].

The roughness of all surfaces was initially examined. All bracket samples were below a threshold value of Ra = 0.2 µm, where the formation of biofilm is not substantially influenced by the surface roughness [8, 52, 62]. The differences in biofilm formation can therefore be attributed solely to the type of layer. For the PIIID-modified samples, the calculated penetration depth of the implanted silver ions was theoretical analyzed, and, contrary to the data provided by the manufacturer, was only in the low nm range. Due to a discussion with the manufacturer one reason for this could be the surface structure of the bracket material, as the roughness prevents the vertical entry of the ion beam into the surface layer and therefore reduces the penetration depth measured perpendicular to the surface. As a result of the calculated low penetration depth of the silver ions, oversaturation with silver ions quickly occurs at the material surface and, as a consequence, silver deposits as an additionally top layer on the surface. In the event of persistent ion radiation, a µm-thick silver top layer forms without increased ion implantation occurring.

As documented in the literature there are position-dependent variations in biofilm coverage within the oral cavity [2, 3]. The fastening of the samples on the occlusal splints was randomized for each test subject with the result that each sample was ultimately fastened to each fixation point at least once. This made it possible to rule out an effect of the sample position. An experiment set-up with splints used to fasten the test specimens has already been used in various studies [3, 21, 29, 43]. The splint was used to protect the enamel, as removing brackets or test specimens fixed directly to the teeth can lead to enamel tears on the surface of the tooth [40, 46, 59]. In contrast to previous studies, shield-like plastic plates were also fitted to the occlusal splint in order to hold off the cheek and tongue [29]. The existing shear forces in the oral cavity could reduce the forming biofilm and therefore falsify the result. The circulation of saliva was not affected by an existing gap between the test specimens and the shield. The period of 48 h, for which the splints were worn, was evaluated in preliminary experiments. This period enables the individually formed initial biofilm to be analyzed in a reproducible manner and has already been selected as the examination period in many other studies [3, 10, 21, 29, 44].

The biofilm growth was analyzed by live/dead fluorescent staining and subsequent CLSM. This well-established method for quantifying initial biofilms enables biofilm morphology to be recorded in an almost native manner [28, 35, 51, 61]. In addition to bacterial cells, in the microscopic image of the stained biofilm, human cells, potentially gingival epithelial cells, can be detected on the samples surfaces. It has already been demonstrated that oral bacteria are able to colonize human gingival epithelial cells and thereby integrate them into the biofilm formation [63]. The quantification of the biofilms showed a significant reduction in plaque accumulation with regard to biofilm volume and surface coverage on all silver-modified surfaces compared to untreated bracket material. Silver has an antibacterial effect due to silver particles inducing destruction of the respiratory chain by inhibiting important enzymes [53]. In addition, they inhibit the DNA replication of the microorganisms [26]. No significant differences between the individual silver surface modifications were observed. In contrast to this, with regard to the live/dead distribution, the PIIID procedure was the only examined surface modification that showed a significant increase in dead bacteria compared to untreated bracket steel and the galvanic coating. This indicates that the implanted silver ions in stainless steel bracket material lead to an improved antimicrobial effect. Therefore, despite the low implantation depth of a few nm, the PIIID procedure presented a significant antibacterial effect.

The bacteria stained with fluorescent dyes show up in color when stimulated accordingly. Bacteria that fluorescent red indicate cells with a destroyed membrane whose nucleic acid has been stained by the penetration of the dye into the cell [11]. Future studies should aim to investigate whether the bacteria dyed red by fluorescent dye can nevertheless achieve growth under optimum oral conditions. If this is the case, it can be assumed that the surface exerts a purely bacteriostatic effect on the biofilm formation. However, if the bacteria cannot multiply even under optimum growth conditions, it can be assumed a bactericidal and therefore more effective action of PIIID silver-modified surfaces. An additional analysis of the composition of the bacteria could also shed light on whether the different silver surface layer modifications lead to a shift in bacterial diversity and potentially to a reduction in pathogenic bacteria species. In addition to the antibacterial effect demonstrated here, the potentially improved abrasion behavior due to the implantation of the silver ions is an advantage of the PIIID procedure. Testing the long-term abrasion resistance of PIIID-modified brackets would be an important future aspect. For this purpose, a splint design without the shield-like plastic plates could be used. Furthermore, higher penetration depths of the silver ions are desirable and could be achieved by expending more energy during the ion bombardment of the bracket material. As silver ions located deep in the material surface would have to have a higher abrasion resistance, a long-lasting antibacterial effect could potentially be expected.

## Conclusion

A comparative analysis of the initial quantitative biofilm formation on bracket material that has been silver-modified in different ways was successfully performed in this study under in situ conditions. The PIIID procedure with silver ions, which was used for the first time in orthodontic materials has a demonstrable antibacterial effect, which is comparable to a PVD or galvanic silver coating with regard to reducing the biofilm volume and the percentage surface coverage. With regard to the live/dead distribution, however, the PIIID is the only surface modification with a significantly higher proportion of dead cells compared to the untreated control and the galvanic coating. It can therefore be concluded that the PIIID procedure has a positive effect with regard to a reduction in initial intraoral biofilm and the live/dead distribution of bacteria and that an investigation into long-term effectiveness and abrasion stability would be of great interest in future studies.
